# Decoding Network Structure in On-Chip Integrated Flow Cells with Synchronization of Electrochemical Oscillators

**DOI:** 10.1038/srep46027

**Published:** 2017-04-07

**Authors:** Yanxin Jia, István Z. Kiss

**Affiliations:** 1 Department of Chemistry, Saint Louis University, 3501 Laclede Av., St. Louis, MO 63103, USA.

## Abstract

The analysis of network interactions among dynamical units and the impact of the coupling on self-organized structures is a challenging task with implications in many biological and engineered systems. We explore the coupling topology that arises through the potential drops in a flow channel in a lab-on-chip device that accommodates chemical reactions on electrode arrays. The networks are revealed by analysis of the synchronization patterns with the use of an oscillatory chemical reaction (nickel electrodissolution) and are further confirmed by direct decoding using phase model analysis. In dual electrode configuration, a variety coupling schemes, (uni- or bidirectional positive or negative) were identified depending on the relative placement of the reference and counter electrodes (e.g., placed at the same or the opposite ends of the flow channel). With three electrodes, the network consists of a superposition of a localized (upstream) and global (all-to-all) coupling. With six electrodes, the unique, position dependent coupling topology resulted spatially organized partial synchronization such that there was a synchrony gradient along the quasi-one-dimensional spatial coordinate. The networked, electrode potential (current) spike generating electrochemical reactions hold potential for construction of an *in*-*situ* information processing unit to be used in electrochemical devices in sensors and batteries.

Networks of discrete units underlie the behavior of evolving systems in engineering (e.g., power grid or internet) and nature (connectonome of the brain)^1^,^2^. There are two fundamental ways network theory can be applied to chemical reactions. A (spatially uniform) collection of chemical reactions can be represented on a graph, where the nodes represent the substances, and the reactions between them are the edges^3^. Graph representation can facilitate fast search for reaction pathways, or identification of positive and negative feedback loops in the mechanism in metabolic and protein interaction networks, or, possibly, in the entire (synthetic) organic chemistry^3^,^4^.

In a different approach, nodes are spatially identifiable units where complex chemical reactions occur and the edges represent interactions. In this dynamical approach, the networks create a new geometrical space for the reaction to take place; this new space holds the promise of generation of novel types of spatiotemporal pattern formation. Small networks can be obtained by mass transfer or electrical coupling of chemical oscillations in tank reactors^5^. However, construction of large chemical networks proved to be a challenge. Large networks are typically constructed externally through a computer feedback^6^ to a population of light sensitive Belousov-Zhabotinsky (BZ) beads system, or a resistor network circuitry^7^ that couples the electrochemical reactions. Such designed networks exhibited chimera states, where synchronized and desynchronized regions co-existed^6^,^8^. In order to obtain inherently coupled network, a promising effort utilized microscale BZ droplets where all the different types of Turing patterns were confirmed in a single setup^9^,^10^.

Microfluidic systems with on-chip integrated electrodes^11^,^12^,^13^, provide a possibility for *in*-*situ* signal processing and computation as long as the rates of the reactions on the electrodes are coupled in network topologies that enable signal processing capabilities. Multiple approaches have been attempted for construction of fluidic networks, in particular, in relation to different types of logic gates. Branched flow channel networks can be designed with fluidic resistance^14^, pneumatics^15^, or surface-tension based passive pumping^16^ to design directed flow in the channels. Microfluidic valves with complex plumbing^17^ or bubble logic^18^ in flow channels can also mimic logic gate design. With electrochemistry, pair-band microelectrodes can create communication channels, in which electrical potential perturbation of one electrode results in electrogeneration of chemical species, which can diffuse to the other electrode for collection and possibly new electrical stimulation^19^. With different mechanism, bipolar electrochemistry^20^,^21^ can be used in a single flow channel to fabricate simple logic gates with inputs as voltage sources and outputs as optical signal from electrogenerated chemiluminescence^22^,^23^.

In this paper, we explore the coupling topology that emerges through the potential drops in the flow channel in a commonly used electrochemical lab-on-chip device, a multielectrode detector system in a microfluidic flow channel. We employ a complex, electrochemical reaction (electrodissolution of nickel)^24^ that generates oscillatory current (and electrode potential) at constant circuit potential. Analysis of the phase of the oscillatory reaction provides an effective means for characterization of the spiking patterns due to the coupling through network topologies. The network topology is decoded using dynamical measurements of reaction rates and phase model analysis (connectomics). The impact of cell geometry on widely different (positive and negative, uni and bidirectional) coupling topology is interpreted with a theoretical model of the potential drop in the flow channel. The effects of the unique, position dependent network topologies on the features of the spatially organized partially synchronized states are analyzed as a function of electrode number (two to six) and cell geometry (position of the electrodes).

## Results

**Figure 1:**
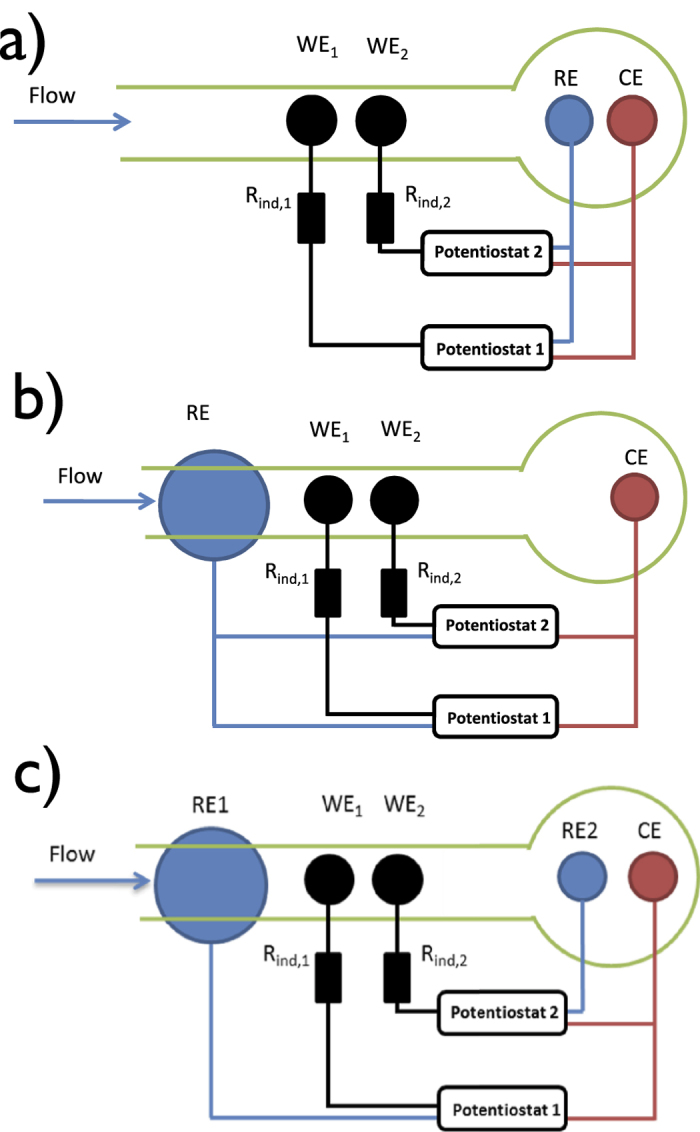
Schematics of three commonly used dual-electrode configurations. (**a**) Traditional (ipsilateral) placement of reference and counter electrodes. (**b**) Upstream (contralateral) reference and counter electrode placements. (**c**) Dual reference electrode configuration. *WE*_1,2_: Ni working electrodes embedded in epoxy; RE and RE2: Ag/AgCl/3 M NaCl reference electrode; CE: Pt counter-electrode. RE1: Ni reference electrode for working electrode 1.

Our general strategy to explore the electrical coupling among the electrode is as follows. First, we consider cell geometries defined as number of working electrodes, and placement of reference/counter electrodes. (Figure 1 shows the schematics of the three considered configurations with two working electrodes). A typical device has a flow channel in which a number of electrodes are placed. The chemical reactions take place on the surfaces of the electrodes, and the rate of the reaction strongly depends on the local electrode potential drop that drives the reaction. The electrochemical reaction generates current, that flows to the counter electrode placed in the reservoir. A potentiostat sets the potentials of the electrodes, such that the potential differences between the working and reference electrodes are constant. Because the current flow between the electrodes introduces potential drops through the electrolyte, the reaction rates measured on the electrodes depend on each other, and the dependence is affected by the spacing of the electrodes and the placement of the counter electrode (which defines the direction of the current) and the reference electrode (which defines a potential with respect to the electrodes are polarized).

Therefore, our next step is the application of equivalent circuit modeling to obtain a mathematical formula for the coupling between the electrodes as a function of the total resistance of the cell and geometry parameters (e.g., resistance, which is proportional to the distance, between the electrodes)^25^,^26^. (Note that because the objective is to explore the effect of electrical coupling between the electrodes, we have chosen a chemical reaction, the electrodissolutin of nickel in sulfuric acid^24^, which is under kinetic control and thus not affected by mass transport effects). Finally, experiments are presented with spiking patterns of the oscillatory reactions to demonstrate the effects of the coupling topology.

The dynamics of the electrode potential of a single electrode in the array can be described with the use of a Randles equivalent circuit. The current (*I*_*k*_) generated by each electrode that has capacitance/surface area *C*_*d*_ is obtained from double layer charging and Faradaic current:


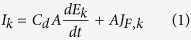


where *A* is electrode surface area, *E*_*k*_ is electrode potential, and *J*_*F*,*k*_ is the Faradayic current density of the k-th electrode. By rearranging equation (1),


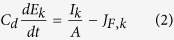


In the presence of many electrodes in the flow channel, the currents generated by the electrodes contribute to the ohmic (IR) potential drops in the flow channel; these ohmic drops affect the electrode potentials of the electrodes in a non-trivial manner and result in network interactions among reactions.

The differential equations for the dynamical evolution of the electrode potentials can be written in a general form as:


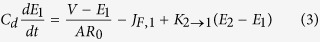



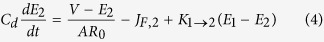


where *V* is circuit potential, and *K*_2→1_ and *K*_1→2_ are the coupling strength from electrode 2 to 1 and electrode 1 to 2, respectively. The derivation of the equations for the considered cell geometries can be found as Supplementary Note. We consider the same circuit potential (*V*) and total cell resistance (*R*_0_) for each electrode; therefore the system parameters for the surface reactions are identical. Such approximation greatly simplifies the extraction of interaction topology between the reaction units.

We assumed that the potential drop in the flow channel is nearly linear and thus the the resistance in the flow channel is proportional to the channel length. In other words, we assume that Ohm’s Law is obeyed for the potential drop in the cell. This assumption was validated in numerical simulations and experimental measurements of the resistance of the flow channel^25^,^27^. Note that such assumption is valid when the quasi-one-dimensinal flow channels are much longer than the size of the electrode; the approximation is usually acceptable in a typical flow channel length of few mm and electrode sizes in the few *μ*m range.

**Figure 2:**
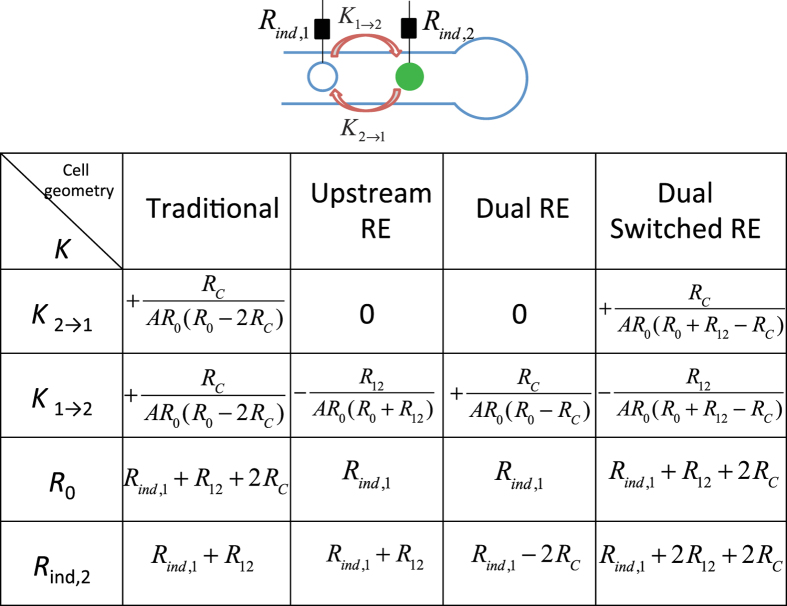
The effect of cell geometry on coupling topology obtained from equivalent circuit analysis of dual working electrodes. RE: reference electrode. *R*_0_: total cell resistance, *R*_*C*_: collective resistance (resistance of electrolyte from working electrode 2 to the reservoir), *R*_12_: solution resistance between two working electrodes, *A*: electrode surface area, *R*_*ind*,1_: external resistance connected to working electrode 1, *R*_*ind*,2_: external resistance connected to working electrode 2. *K*: coupling strength. (The derivations of these equations can be found as Supplementary Note).

The coupling strengths obtained from equivalent circuit analysis between two working electrodes are given in Fig. 2.

### Ipsilateral (traditional) placement configuration

First we consider two working electrodes in the flow channel at ipsilateral (traditional) placement in which the reference and counter electrodes are at the same downstream end of the flow channel as shown in Fig. 1(a).

The theoretical analysis (see Fig. 2) showed that 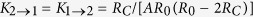. The dynamics is affected by bidirectional positive coupling that depends on the total cell resistance *R*_0_, distance from downstream electrode to the reservoir (through solution collective resistance *R*_*C*_), and electrode surface area *A*. Thus, the mathematical analysis suggest the existence of bidirectional positive coupling within the cell.

**Figure 3:**
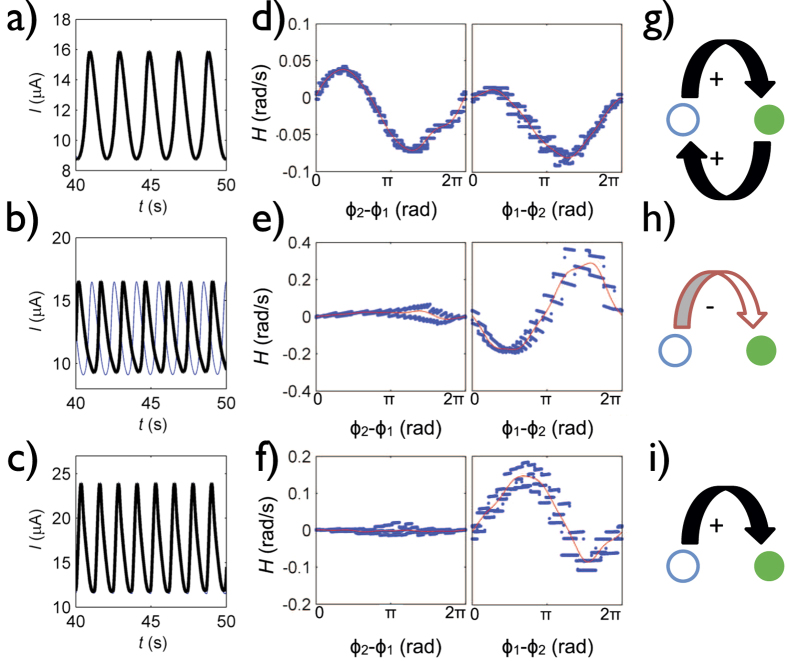
Effects of cell geometry on synchronization of current oscillations between two working electrodes. Top row: two electrodes with ipsilateral (traditional) placement of reference and counter electrodes. The distance from the downstream electrode to the reservoir *D*_2*R*_ = 5.0 mm, the distance between the two electrodes *D*_12_ = 2.4 mm. *V* = 1.68 V. Middle row: Dual electrodes with reference and counter electrodes at opposite sides. *D*_2*R*_ = 6.0 mm, *D*_12_ = 6.1 mm. *V* = 1.79 V. Bottom row: dual-reference electrode system. *D*_2*R*_ = 1.5 mm, *D*_12_ = 12 mm. *V* = 1.74 V. (**a**–**c**) Current vs. time plots. (**d**–**f**) Phase interaction functions for electrode 1 (left) and 2 (right). (**g**–**i**) Schematics of coupling topologies. The open and shaded circles represent the electrodes (corresponding to the schematics in Fig. 1). Arrows and the +/− symbols represent the direction and sign of the coupling, respectively. For all experiments the total cell resistance *R*_0_ = 20 kΩ.

We performed experiments in a dual electrode setup to confirm the theoretical findings. In this configuration, the oscillatory metal dissolution of nickel in sulfuric acid exhibited in-phase synchrony (see Fig. 3(a)).

With an oscillatory system close to the onset of oscillations, the synchronization pattern is typically either in in-phase or anti-phase configuration. Positive coupling implies that electrode potential difference will create a cross-current between the electrodes, which diminishes the potential difference between the electrodes. Therefore, very often positive coupling between the electrodes will result in in-phase configuration. Indeed, in previous investigations with macrocells, where positive coupling was induced with an external resistance interface, in-phase synchrony behavior was observed^28^. In contrast, when negative coupling is present (e.g., with IR compensation), anti-phase oscillations can occur^28^. The in-phase synchronized oscillations (shown in Fig. 3(a) in the microchip setup) thus confirms the presence of positive coupling. The observation of in- or anti-phase behavior cannot differentiate between symmetrical (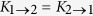) and unidirectional (e.g., *K*_1→2_ = 0, 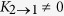) coupling topologies.

Extracting coupling topology from a network of dynamical units is a challenging task that requires advanced signal processing techniques^29^,^30^. Here we apply a method that uses phase models to characterize the interaction topology^31^,^32^. For uncoupled elements, the phase of the oscillations (*ϕ*) increases linearly with time at a rate of the natural frequency (*ω*), i.e., *dϕ*/*dt* = *ω*. However, when the oscillations are coupled, there will be deviations from the linear variations: the deviations depend on the phase difference between the coupled elements, therefore, we can express the rate of phase change with the following equations^31^:


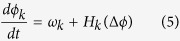


where *t* is time, *ϕ*_*k*_ and *ω*_*k*_ (*k* = 1, 2) are phases and natural frequencies of the oscillations, Δ*ϕ* is the phase difference, and *H*_*k*_ are the phase interaction functions.

The interaction functions *H*_*k*_ can be determined by plotting d*ϕ*_*k*_/d*t* − *ω*_*k*_ vs. Δ*ϕ* from an experiment with phase slipping behavior^32^. As shown in Fig. 3(d), the interaction functions for for both units are nearly sinusoidal functions. Coupling topology extraction is based on the assumption that the coupling strength is proportional to the amplitude of the interaction function. Therefore, when the amplitude of the interactions functions of two dynamically identical units are the same, the coupling is symmetrical. Conversely, with only unidirectional coupling the phase dynamics of the driven node is affected, i.e., the amplitude of the interaction function of the driver node must be zero. The symmetrical and unidirectional couplings represent two extremes by which two units can interact. The coupling between two units can be described by the coupling strengths in both directions (i.e., *K*_1→2_ and *K*_2→1_), or by a combination of a mean coupling strength and a directionality index, 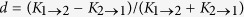
31. We can obtain the directionality index (*d*) from the comparison of the amplitudes of the (first) Fourier harmonics (*c*_*k*_) of the interaction functions *H*_*k*_: 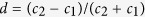. *d* = ±1 and 0 indicate strictly unidirectional and bidirectional coupling, respectively. The interaction functions for the two electrodes at ipsilateral (traditional) placement (Fig. 3(d)) show approximately positive sinusoidal functions with amplitudes *c*_1_ = 0.05 rad/s, *c*_2_ = 0.04 rad/s (for electrodes 1 and 2, respectively), therefore, the analysis supports the presence of positive bidirectional (*d* = 0.1) coupling between the electrodes (shown in Fig. 3(g)).

### Contralateral placement configuration

In another configuration with contralateral placement of reference/counter electrodes, the reference and counter electrodes are placed at the opposite end of the flow channel where the two working electrodes reside (see Fig. 1(b)).

The corresponding equivalent circuit analysis (Fig. 2) shows that there is no coupling from the downstream to the upstream electrode and the coupling from the upstream to the downstream electrode is a negative value that depends on the resistance (and thus the distance) between two working electrodes *R*_12_, i.e., *K*_2→1_ = 0, 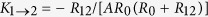.

Experiments with contralateral placement configuration were carried out with a dual electrode system, where a 2 mm diameter Ni electrode placed upstream to the working electrodes served as a (pseudo) reference electrode. In this configuration the observed anti-phase oscillations (Fig. 3(b)) indicate the presence of negative coupling. The phase interaction function (Fig. 3(e)) is a negative sinusoidal function with amplitude *c*_2_ = 0.23 for the downstream electrode and a nearly straight line with negligible amplitude *c*_1_ = 0.01 for the upstream electrode. Consequently, unidirectional coupling (schematically shown in Fig. 3(h)) is quantified by the large value of the directionality index *d* = 0.9. Note that by a simple movement of the reference electrode from the downstream to the upstream positions changed the sign and directionality of the coupling in the network.

### Dual-reference electrode configuration

In a third arrangement (dual-reference electrode system, see Fig. 1(c)), the upstream 2 mm diameter nickel electrode serves as the reference electrode for upstream working electrode (WE1), the downstream Ag/AgCl/3 M NaCl reference electrode serves as the reference point for downstream working electrode (WE2), and both of the working electrodes share the same 0.5 mm thick Pt wire counter electrode at the downstream end of the flow channel.

In this configuration, the equivalent circuit analysis indicated that there is a unidirectional positive coupling that depends on the distance from downstream electrode to the reservoir, i.e., *K*_2→1_ = 0 and 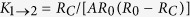. Thus by changing the position and number of reference electrodes, the coupling topology can be tuned between bidirectional positive, unidirectional negative, and unidirectional positive.

With this setup, in-phase oscillations (Fig. 3(c)) were observed, which is an indication of positive coupling. Although the in-phase synchronization would imply some resemblance to the first (traditional) configuration with bidirectional positive coupling, the phase interaction function analysis (see Fig. 3(f)) indicates that only the downstream electrode is affected by coupling (*c*_1_ = 0.00, *c*_2_ = 0.10), and thus the coupling is unidirectional (*d* = 1.0), as shown in Fig. 3(i).

### Switched dual-reference electrode configuration

**Figure 4:**
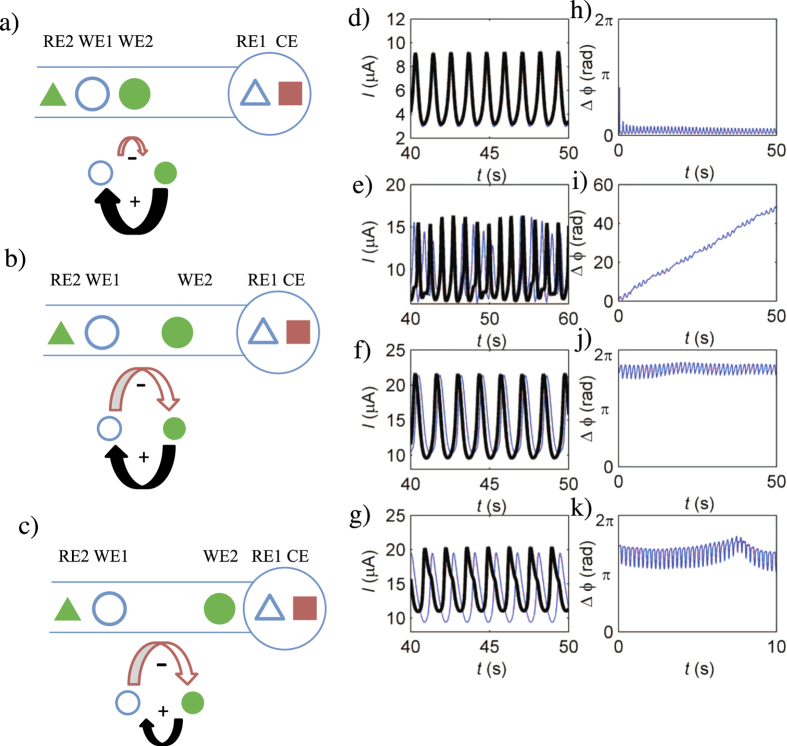
Effect of placement of the middle working electrode on synchronization and network topology in switched dual reference electrode setup. Top row: Distant WE2-RE1 placement (*D*_12_ = 0.5 mm, *D*_2*R*_ = 15.5 mm). Middle row: medium placement (*D*_12_ = 5.4 mm, *D*_2*R*_ = 5.8 mm). Bottom row: close placement (*D*_12_ = 10.0 mm, *D*_2*R*_ = 0.7 mm). (**a**–**c**) Schematic diagram of dual electrode cell (top) and coupling topology (bottom). WE1,2: Ni working electrodes, RE1: Ag/AgCl/3 M NaCl reference electrode for WE1, RE2: Ni reference electrode for WE2, CE: Pt counter electrode. (**d**–**g**) current vs. time plots. *R*_0_ = 20 kΩ. *V* = 1.61 V, 1.64 V, 1.62 V and 1.69 V respectively. (**h**–**k**) phase difference vs. time plot.

Alternative cell geometry was studied with the dual-reference electrode system, but the corresponding reference electrodes of individual working electrodes were exchanged (switched dual-reference electrode configuration, Fig. 4(a)). In this configuration, the upstream nickel electrode serves as the reference electrode for downstream working electrode (WE2), the downstream Ag/AgCl/3 M NaCl reference electrode serves as the reference electrode for upstream working electrode (WE1).

There is a positive coupling in one direction (downstream to upstream working electrode) and a negative coupling in the other direction, i.e., 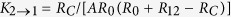, 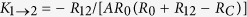 (see Fig. 2).

With a simple change of the position of the downstream electrode the dominating positive (Fig. 4(a)) or negative (Fig. 4(c)) coupling can cause in-phase (Fig. 4(d,h)) or out-of-phase (Fig. 4(g,k)) synchrony, respectively. In configurations where the positive and negative couplings had comparable magnitudes (Fig. 4(b)) in-phase synchrony was difficult to achieve: in some experiments complex oscillations with 4:5 entrainment behavior (Fig. 4(e,i)), and in other experiments slightly out of phase synchronized oscillations were observed (Fig. 4(f,j)). The experiments thus show a potential for creating a largely different coupling directionality and strength by a simple change of the working electrode position WE2.

### Configuration with three and six working electrodes

**Figure 5:**
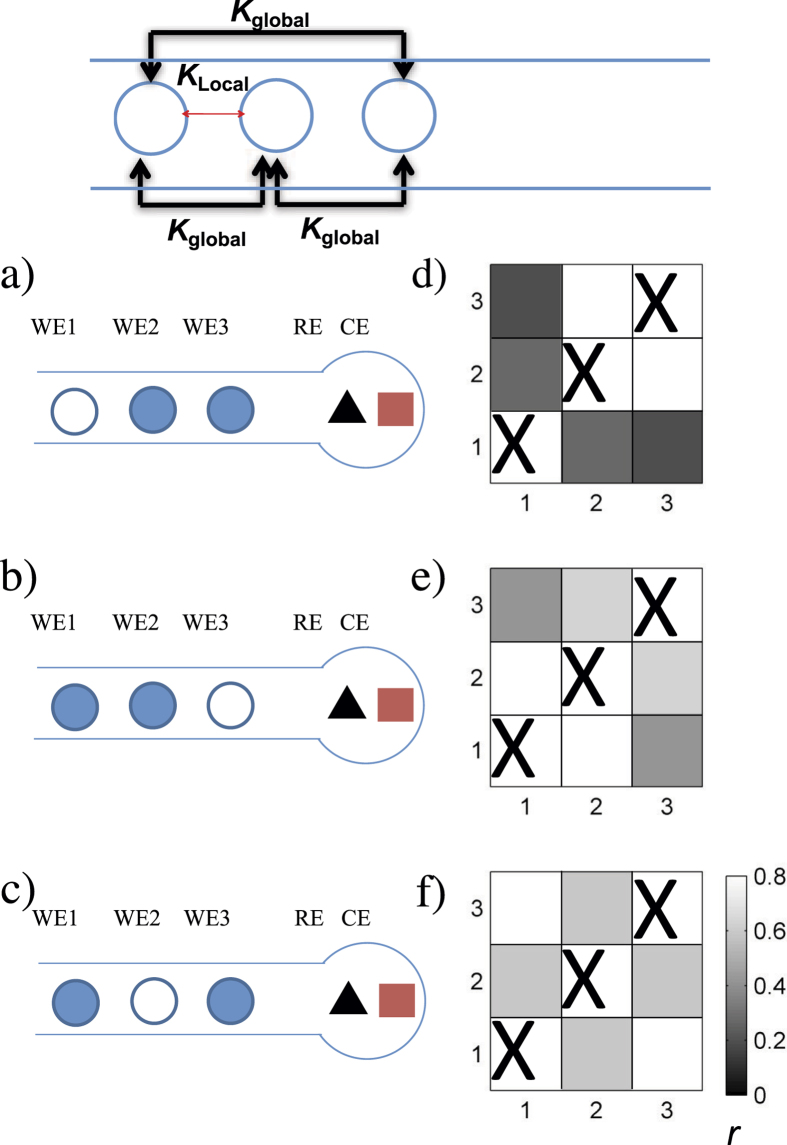
Three electrode networks with large distance to reservoir: dominating global coupling and partial synchronization without spatial organization. Top: coupling topology. (**a**–**c**) Schematics of cells: shaded circles denote synchronized electrodes. (**d**–**f**) Synchronization matrices for the corresponding experiments. (**a**) *D*_12_ = 2.9 mm, *D*_23_ = 3.0 mm, *D*_3*R*_ = 11.9 mm, *R*_0_ = 100 kΩ, *V* = 2.30 V. (**b**) *D*_12_ = 3.7 mm, *D*_23_ = 1.4 mm, *D*_3*R*_ = 12.4 mm, *R*_0_ = 120 kΩ, *V* = 2.55 V. (**c**) Same parameters as in panel (b) except for *V* = 2.45 V.

With the use of more than two electrodes, a network of reaction units could be obtained. Here we consider only traditional placements of reference and counter electrodes placed at the end of the flow channel as shown in the schematics in Fig. 5(a). Based on theoretical circuit analysis (see Supporting Note), the network topology consists of global coupling 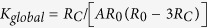 between each electrode pairs and an additional local coupling 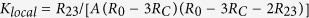 between the two upstream electrodes, where *R*_*C*_ is the solution collective resistance (depending on distance from electrode 3 to the reservoir), and *R*_23_ is the solution resistance between working electrodes 2 and electrode 3. The definitions of *K*_*global*_ and *K*_*local*_ show that the coupling strengths between each electrode can be tuned by changing *R*_23_ and *R*_*C*_.

The experiments were carried out with three micro-wires as shown in Fig. 5(a). Here we consider intermediate coupling strength at which the current oscillations of two out of three electrodes are fully synchronized, while third electrode is in the phase slipping state. To characterize the extent of synchrony between a pair of oscillators we use an order parameter *r* based on phase difference^33^ defined as


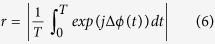


where *j* is the imaginary unit, and *T* is the averaging time. (*r* = 0 and 1 indicate no or complete synchrony, respectively).

When the distance between WE2 and WE3 (*D*_23_) is smaller than the distance between WE3 and the reservoir (*D*_3*R*_), the global coupling will drive the synchrony; the coupling topology is shown in the top panel of Fig. 5. Theoretical expectation of globally coupled population of oscillators in the partially synchronized state is that the identity of the synchronized oscillators will be uniquely determined by their natural frequencies^34^, thus independent of their geometrical positions. We performed a series of experiments with different electrodes when *D*_23_ < *D*_3*R*_; representative experiments are shown in Fig. 5(a–c). As shown in the synchronization matrices in Fig. 5(d–f), when the oscillators exhibited partial synchrony, the two synchronized oscillators could occur from any possible pairs (1-2, 1-3, or 2-3), thus the synchrony pattern does not depend on the electrode placement. These results are in agreement with global coupling topology: partial synchrony does not exhibit spatial order.

**Figure 6:**
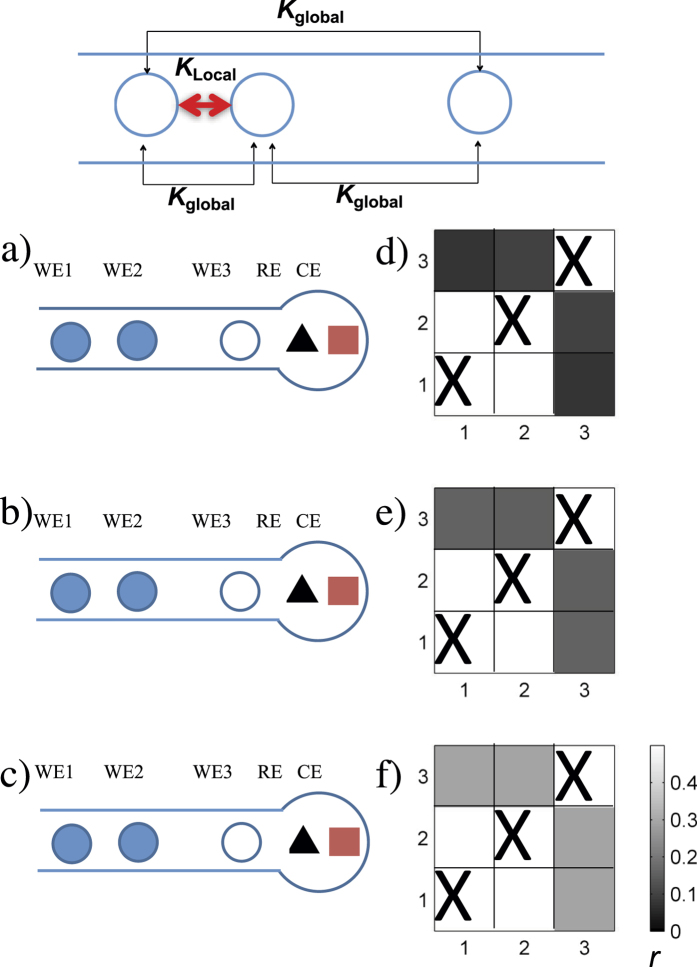
Three electrode networks with close placement to reservoir: dominating upstream coupling and spatially organized partial synchronization. Top: coupling topology. (**a**–**c**) Schematics of cells: Shaded circles denote synchronized electrodes. (**d**–**f**) Synchronization matrices for the corresponding experiments. (**a**) *D*_12_ = 0.5 mm, *D*_23_ = 5.8 mm, *D*_3*R*_ = 1.2 mm, *R*_0_ = 50 kΩ, *V* = 1.93 V. (**b**) *D*_12_ = 1.8 mm, *D*_23_ = 4.2 mm, *D*_3*R*_ = 1.0 mm, *R*_0_ = 20 kΩ, *V* = 1.68 V. (**c**) *D*_12_ = 1.8 mm, *D*_23_ = 2.9 mm, *D*_3*R*_ = 0.8 mm, *R*_0_ = 20 kΩ, *V* = 1.6 V.

When the distance between WE2 and WE3 (*D*_23_) is larger than the distance between WE3 and reservoir (*D*_3*R*_), the local (upstream) coupling between WE1 and WE2 will drive the synchrony; the coupling topology is shown in the top panel of Fig. 6. Therefore, it is expected that the two upstream electrodes will be more synchronized than the other electrode pairs. To confirm this expectation, we repeated several experiments when *D*_23_ > *D*_3*R*_ (see Fig. 6(a–c)). The synchronization matrices in Fig. 6(d–f) always showed that the two upstream electrodes (1-2) are more synchronized than the other electrode pairs. We refer to this state as spatially organized partial synchrony (SOPS).

**Figure 7:**
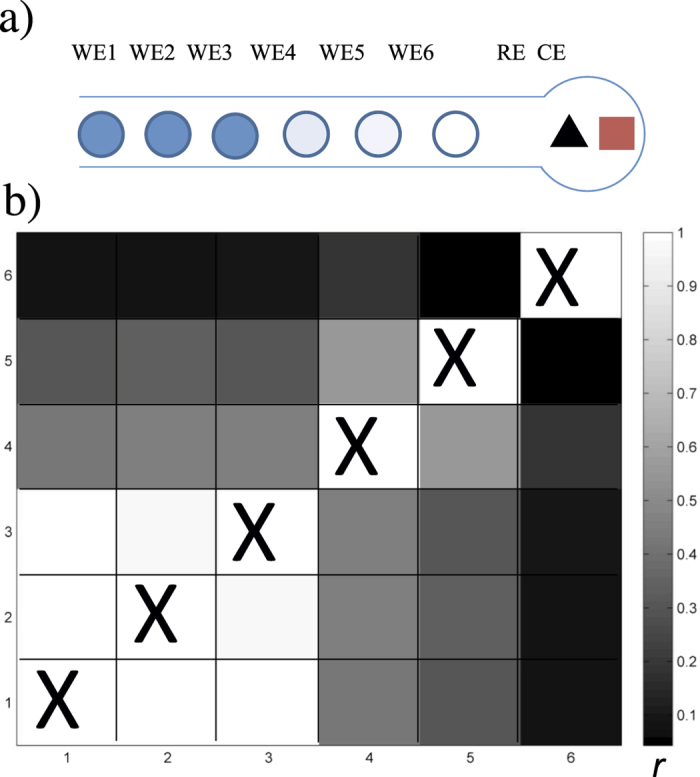
Spatially organized partially synchronized pattern with synchrony gradient in a six-electrode cell. (**a**) Schematic of the cell (the spacing of the electrodes is approximately 2 mm). (**b**) Synchronization matrix. *V* = 1.95 V, *R*_0_ = 50 kΩ.

As an extension of the spatial pattern shown before, a set of electrodes in the flow channel would be expected to have strong coupling among upstream electrodes and less coupling among downstream electrodes. In Fig. 7, we show the results obtained with six approximately equally spaced electrodes in the partially synchronized state. The three upstream electrodes (electrodes 1, 2 and 3) are synchronized with same frequency (0.476 Hz), the downstream electrodes are less synchronized (electrodes 4 and 5 with frequency of 0.520 Hz and 0.534 Hz, respectively), and electrode 6 is not synchronized with even larger frequency (0.601 Hz) as shown in Fig. 7. Thus, the unique, position dependent network topology imposes a dynamical behavior with a gradient synchrony; such SOPS behavior seems to be the general feature of the linear array of microelectrochemical oscillator system.

## Discussion

We demonstrated that the electrical coupling between the electrodes can generate networks with a variety of different topologies in microfluidic devices. Chemical connectomics, the decoding of network topology from dynamical measurements, was proven to be a useful tool to verify the predicted connectivities in two-unit systems. (For larger systems, advanced signal processing could be applied that considers indirect interactions^35^). The mathematical formulas obtained for the coupling strengths hold promise for estimating the electrical cross-coupling in microfluidic devices in practical applications. In electroanalytical applications using traditional configurations, where the reference and counter electrodes are placed in a reservoir at the end of the flow channel, the distance between the most downstream electrode to the reservoir should be minimized to avoid global coupling. Attempts to eliminate coupling by moving the reference electrode to upstream position certainly facilitates the control of electrode potential of the upstream electrode, however, this configuration introduces dangerous negative (unidirectional) coupling between the electrodes. When this negative coupling is strong, it could create highly nonuniform reaction rates on the electrode surface, in particular, with reactions with negative slopes in the current vs. potential characteristics^36^. Therefore, when electrode arrays are employed, e.g., with scanning electrochemical microscopy^37^, care must be taken to allow current to disperse in the cell (e.g., by parallel placement of counter and working electrodes) to eliminate electrical coupling. The electrical coupling can be most adverse with generator-collector type of devices in linear flow channels^11^; in thise case, small electrode areas and addition of parallel resistors could minimize the coupling effects.

The micro-electrochemical system studied here can accommodate large number of electrodes, and thus could represent an important experimental system for large set of networked chemical reactions. With the use of multichannel potentiostats, multiple groups of working electrodes could form modular networks, and the inter and intra-group coupling could be controlled by the application of multiple reference electrodes assigned to each groups. Such complex networks could be designed for specific applications. For example, a network with positive and negative connections can be designed with oscillatory units for associate memory arrays that can be used for pattern recogniation and storage^38^. By application of such information processing, the device could be integrated with traditional microfluidic sensors and batteries to create an all-electrochemical intelligent sensor device.

## Methods

The experimental system is a microfluidic flow reactor in which 100 *μ*m diameter nickel electrodes (embedded in epoxy) are sealed with polydimethylsiloxane chip. 2 M sulfuric acid/0.01 M NiSO_4_ electrolyte is pumped in a 200 *μ*m (width) × 100 *μ*m (height) flow channel at flow rate Q = 1.5 *μ*L/min that corresponds to a linear velocity of 0.125 cm/s. The Ag/AgCl/3 M NaCl reference electrode (exhibiting 209 mV electrode potential vs. standard hydrogen electrode) and a 0.5 mm thick Pt wire counter electrode are placed into the center of the reservoir. Further details about the the construction of the cell are given in a previous publications^25^,^26^.

When a single electrode is present in the flow channel and the electrode is polarized at a constant potential 1.6 V (vs. the reference electrode) the electrode reactions generate oscillatory dissolution rate (measured as the current) due to the secondary passivation of nickel^25^,^26^. The oscillation occur because of the interactions of a fast positive feedback loop and a delayed negative feedback loop in the reaction mechanism. Because of the negative differential resistance (decreasing current with increasing electrode potential), when the electrode potential increases, the current, and thus the ohmic (IR) potential drop in the cell decreases; as the circuit potential is kept constant, the electrode potential will thus increase which results in further decrease of the current. This fast positive feedback loop is then followed by delayed response of relaxation of oxide film coverage to steady state value; because the relaxation is relatively slow process compared to the time-scale of the variation of electrode potential, the feedback loops generate oscillatory metal dissolution. (We note that although we investigate the process in the oscillatory region, the derived networks are inherent properties of the cell configurations; we chose oscillatory reactions because with these reactions the dynamical behavior is is strikingly different for the different networks).

With more than one electrode, each of the electrodes exhibited smooth current oscillations (nearly sinusoidal waveform close to the Hopf bifurcation point) obtained at constant circuit potentials with 30 mV above Hopf bifurcation. (Because the onset of oscillations occurs at slightly different potentials for each experiment, we report the circuit potentials for every experiment).

The schematics of electrochemical cells are shown in Fig. 1(a–c). Ipsilateral (traditional) placement of reference/counter electrodes (Fig. 1(a)) refers to the reference electrode and counter electrodes positioned at the same downstream end of the flow channel. For contralateral placement of reference/counter electrodes, the reference electrode is placed at the upstream end of the flow channel (Fig. 1(b)). A 2 mm diameter Ni electrode, placed upstream to the working electrodes, serves as a reference electrode and a 0.5 mm thick Pt wire serves as counter electrode. Dual-reference electrode system is shown in Fig. 1(c), where the upstream 2 mm diameter nickel electrode serves as the reference electrode for upstream working electrode (WE1), the downstream Ag/AgCl/3 M NaCl reference electrode serves as the reference point for downstream working electrode (WE2), and both of the working electrodes share the same 0.5 mm thick Pt wire counter electrode. The switched dual-reference electrode configuration refers to cell that the upstream nickel electrode serves as the reference electrode for downstream working electrode (WE2), the downstream Ag/AgCl/3 M NaCl reference electrode serves as the reference electrode for upstream working electrode (WE1). Note that once the electrodes are integrated in the flow channel, it is very easy to switch between the different configurations by connecting the corresponding working, reference, and counter electrodes to the potentiostat.

## Additional Information

**How to cite this article:** Jia, Y. and Kiss, I.1 Z. Decoding Network Structure in On-Chip Integrated Flow Cells with Synchronization of Electrochemical Oscillators. *Sci. Rep.* **7**, 46027; doi: 10.1038/srep46027 (2017).

**Publisher's note:** Springer Nature remains neutral with regard to jurisdictional claims in published maps and institutional affiliations.

## Supplementary Material

Supplementary Information

## References

[b1] Albert R. & Barabasi A. L. Statistical mechanics of complex networks. Rev. Mod. Phys. 74, 47–97 (2002).

[b2] Newman M. E. J. The structure and function of complex networks. SIAM Rev. 45, 167–256 (2003).

[b3] Eiswirth M., Freund A. & Ross J. Mechanistic classification of chemical oscillators and the role of species. Adv. Chem. Phys. 80, 127–199 (1991).

[b4] Fialkowski M., Bishop K. J. M., Chubukov V. A., Campbell C. J. & Grzybowski B. A. Architecture and evolution of organic chemistry. Angew. Chem. Int. Ed. 44, 7263–7269 (2005). 10.1002/anie.200502272 16276556

[b5] Epstein I. R. & Showalter K. Nonlinear chemical dynamics: Oscillations, patterns, and chaos. J. Phys. Chem. 100, 13132–13147 (1996).

[b6] Tinsley M. R., Nkomo S. & Showalter K. Chimera and phase-cluster states in populations of coupled chemical oscillators. Nat. Phys. 8, 662–665 (2012). 10.1103/PhysRevLett.110.244102 25165927

[b7] Wickramasinghe M. & Kiss I. Z. Spatially organized dynamical states in chemical oscillator networks: synchronization, dynamical differentiation, and chimera patterns. PloS One 8, e80586 (2013). 24260429 10.1371/journal.pone.0080586 PMC3829877

[b8] Wickramasinghe M. & Kiss I. Z. Spatially organized partial synchronization through the chimera mechanism in a network of electrochemical reactions. Phys. Chem. Chem. Phys. 16, 18360–18369 (2014). 25069401 10.1039/c4cp02249a

[b9] Toiya M., Vanag V. K. & Epstein I. R. Diffusively coupled chemical oscillators in a microfluidic assembly. Angew. Chem., Int. Ed. 47, 7753–7755 (2008). 10.1002/anie.200802339 18756573

[b10] Tompkins N. . Testing turing’s theory of morphogenesis in chemical cells. PNAS 111, 4397–4402 (2014). 24616508 10.1073/pnas.1322005111 PMC3970514

[b11] Amatore C., Da Mota N., Sella C. & Thouin L. General concept of high-performance amperometric detector for microfluidic (bio)analytical chips. Anal. Chem. 80, 4976–4985 (2008). 18470995 10.1021/ac800227t

[b12] Roston D. A. & Kissinger P. T. Series dual-electrode detector for liquid chromatography/electrochemistry. Anal. Chem. 54, 429–434 (1982).

[b13] Martin R. S., Gawron A. J., Lunte S. M. & Henry C. S. Dual-electrode electrochemical detection for poly(dimethylsiloxane)-fabricated capillary electrophoresis microchips. Anal. Chem. 72, 3196–3202 (2000). 10939387 10.1021/ac000160t

[b14] Oh K. W., Lee K., Ahn B. & Furlani E. P. Design of pressure-driven microfluidic networks using electric circuit analogy. Lab Chip 12, 515–545 (2012). 22179505 10.1039/c2lc20799k

[b15] Grover W. H., Ivester R. H. C., Jensen E. C. & Mathies R. A. Development and multiplexed control of latching pneumatic valves using microfluidic logical structures. Lab Chip 6, 623 (2006). 16652177 10.1039/b518362f

[b16] Toepke M. W., Abhyankar V. V. & Beebe D. J. Microfluidic logic gates and timers. Lab Chip 7, 1449 (2007). 17960270 10.1039/b708764k

[b17] Kou S. . Fluorescent Molecular Logic Gates Using Microfluidic Devices. Angew. Chem. Int. Edit. 47, 872–876 (2008). 10.1002/anie.200703813 17943951

[b18] Prakash M. & Gershenfeld N. Microfluidic bubble logic. Science 315, 832–835 (2007). 17289994 10.1126/science.1136907

[b19] Amatore C., Thouin L. & Warkocz J. S. Artificial neurons with logical properties based on paired-band microelectrode assemblies. Chem. Eur. J. 5, 456–465 (1999).

[b20] Chang B. Y., Crooks J. A., Chow K. F., Mavre F. & Crooks R. M. Design and operation of microelectrochemical gates and integrated circuits. J. Am. Chem. Soc. 132, 15404–15409 (2010). 20942419 10.1021/ja107095z

[b21] Zhan W. & Crooks R. M. Microelectrochemical Logic Circuits. J. Am. Chem. Soc. 125, 9934–9935 (2003). 12914451 10.1021/ja0366585

[b22] Chow K. F., Mavre F. & Crooks R. Wireless electrochemical dna microarray sensor. J. Am. Chem. Soc. 130, 7544–7545 (2008). 18505258 10.1021/ja802013q

[b23] Chang B. Y., Chow K. F., Crooks J. A., Mavré F. & Crooks R. M. Two-channel microelectrochemical bipolar electrode sensor array. Analyst 137, 2827–2833 (2012). 22576232 10.1039/c2an35382b

[b24] Lev O., Wolffberg A., Pismen L. M. & Sheintuch M. The structure of complex behavior in anodic nickel dissolution. J. Phys. Chem. 93, 1661–1666 (1989).

[b25] Cioffi A. G., Martin R. S. & Kiss I. Z. Electrochemical oscillations of nickel electrodissolution in an epoxy-based microchip flow cell. J. Electroanal. Chem. 659, 92–100 (2011). 10.1016/j.jelechem.2011.05.007 PMC3150514 21822407

[b26] Jia Y. & Kiss I. Z. Spontaneously synchronized electrochemical micro-oscillators with nickel electrodissolution. J. Phys. Chem. C 116, 19290–19299 (2012).

[b27] Birzu A., Jia Y., Sankuratri V., Liu Y. & Kiss I. Z. Spatially Distributed Current Oscillations with Electrochemical Reactions in Microfluidic Flow Cells. Chem Phys Chem 16, 555–566 (2015). 25598243 10.1002/cphc.201402631

[b28] Kiss I. Z., Zhai Y. M. & Hudson J. L. Predicting mutual entrainment of oscillators with experiment-based phase models. Phys. Rev. Lett. 94, 248301 (2005). 16090583 10.1103/PhysRevLett.94.248301

[b29] Timme M. & Casadiego J. Revealing networks from dynamics: an introduction. J. Phys. A: Math. Theor. 47, 343001 (2014).

[b30] Angulo M. T., Moreno J. A., Lippner G., Barabási A.-L. & Liu Y.-Y. Fundamental limitations of network reconstruction from temporal data. J. R. Soc. Interface 14, 20160966 (2017). 28148769 10.1098/rsif.2016.0966 PMC5332581

[b31] Kralemann B., Cimponeriu L., Rosenblum M., Pikovsky A. & Mrowka R. Uncovering interaction of coupled oscillators from data. Phys. Rev. E 76, 055201 (2007). 10.1103/PhysRevE.76.055201 18233706

[b32] Tokuda I. T., Wickramasinghe M. & Kiss I. Z. Detecting connectivity of small, dense oscillator networks from dynamical measurements based on a phase modeling approach. Phys. Lett. A 377, 1862–1867 (2013).

[b33] Schelter B., Winterhalder M., Dahlhaus R., Kurths J. & Timmer J. Partial phase synchronization for multivariate synchronizing systems. Phys. Rev. Lett. 96, 208103 (2006). 16803212 10.1103/PhysRevLett.96.208103

[b34] Kuramoto Y. Chemical Oscillations, Waves and Turbulence (Springer, Berlin, 1984).

[b35] Nawrath J. . Distinguishing direct from indirect interactions in oscillatory networks with multiple time scales. Phys. Rev. Lett. 104, 038701 (2010). 20366687 10.1103/PhysRevLett.104.038701

[b36] Jain S., Kiss I. Z., Breidenich J. & Hudson J. L. The effect of IR compensation on stationary and oscillatory patterns in dual-electrode metal dissolution systems. Electrochim. Acta 55, 363–373 (2009).

[b37] Barker A. L., Unwin P. R., Gardner J. W. & Rieley H. A multi-electrode probe for parallel imaging in scanning electrochemical microscopy. Electrochem. Comm. 6, 91–97 (2004).

[b38] Nishikawa T., Lai Y. & Hoppensteadt F. Capacity of oscillatory associative-memory networks with error-free retrieval. Phys. Rev. Lett. 92, 108101 (2004). 15089247 10.1103/PhysRevLett.92.108101

