# Supplementary material for: Decoding Network Structure in On-Chip Integrated Flow Cells with Synchronization of Electrochemical Oscillators
Source: Sci Rep. 2017 Apr 7;7:46027. doi: 10.1038/srep46027 (PMC5384074; doi:10.1038/srep46027)
Supplement: Supplementary Information [file srep46027-s1.pdf]

# Supplementary Information
# Decoding Network Structure in On-Chip Integrated Flow Cells with Synchronization of Electrochemical Oscillators

Yanxin Jia and István Z. Kiss

*Department of Chemistry, Saint Louis University,*

*3501 Laclede Av., St. Louis, MO 63103*

**Supplementary Note: Equivalent circuit analyses**

The working electrodes coupled through the potential drop in the electrolyte can be modeled with the use of a Randles equivalent circuit. The current ($I_k$) generated by each electrode that has capacitance / surface area $C_d$ is obtained from double layer charging and Faradaic current:

$$I_k = C_d A \frac{dE_k}{dt} + A J_{F,k} \tag{1}$$

where $A$ is electrode surface area, $E_k$ is electrode potential, and $J_{F,k}$ is the Faradayic current density of the k-th electrode. By rearranging equation 1,

$$C_d \frac{dE_k}{dt} = \frac{I_k}{A} - J_{F,k} \tag{2}$$

## A. Ipsilateral (traditional) placement configuration

At ipsilateral (traditional) placement of reference / counter electrodes (see Fig. S1), the two electrodes are connected to the potentiostat through individual resistors ($R_{ind,1}$ and $R_{ind,2}$). The current of the upstream electrode ($I_1$) flows in the channel to the downstream electrode (electrode 2) through resistive circuit element $R_{12}$. In the channel below the downstream electrode the total current ($I_1$+$I_2$) flows through resistance $R_C$. The potentiostat maintains constant circuit potential $V$, therefore:

For upstream electrode

$$V = I_1(R_{ind,1} + R_{12}) + (I_1 + I_2)R_C + E_1 \tag{3}$$

For downstream electrode

$$V = I_2 R_{ind,2} + (I_1 + I_2)R_C + E_2 \tag{4}$$

We set $R_{ind,2} = R_{ind,1} + R_{12}$.

By rearranging equation 3 and 4,

$$I_1 = \frac{(V - E_1)(R_{ind,1} + R_{12}) + R_C(E_2 - E_1)}{(R_{ind,1} + R_{12})(R_{ind,1} + R_{12} + 2R_C)} \tag{5}$$

$$I_2 = \frac{(V - E_2)(R_{ind,1} + R_{12}) + R_C(E_1 - E_2)}{(R_{ind,1} + R_{12})(R_{ind,1} + R_{12} + 2R_C)} \tag{6}$$

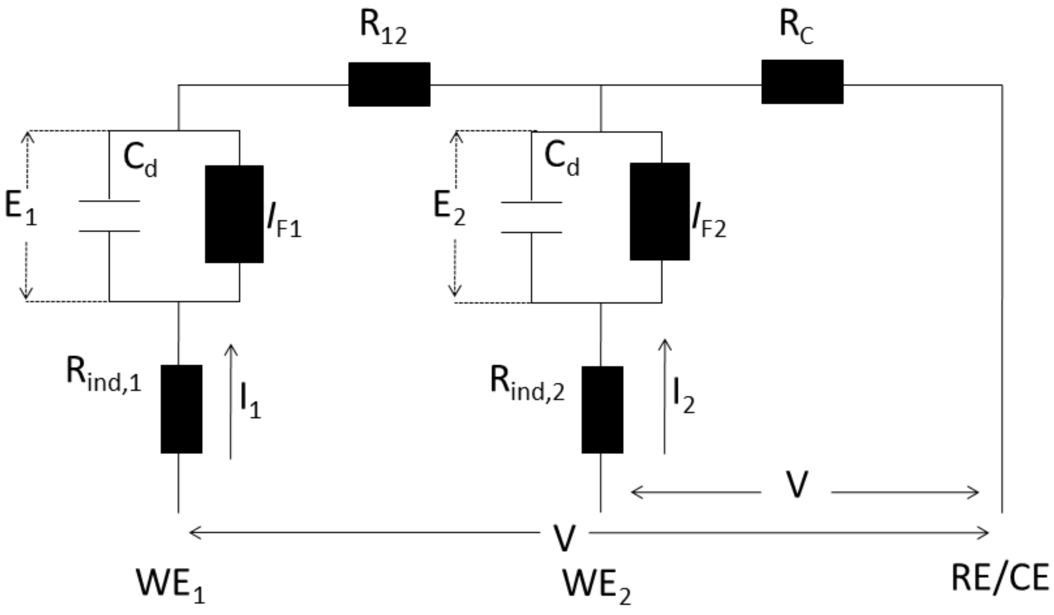


FIG. S1. The equivalent circuit of a dual-electrode electrochemical cell at ipsilateral (traditional) placement of reference (RE) and counter (CE) electrodes, $E_{1,2}$: electrode potential, $I_{F1,2}$: Faradaic current, $C_d$: double-layer capacitance, $R_{ind,1,2}$: individual resistors, $R_{12}$: solution resistance between working electrodes, $I_1$ and $I_2$: current. $R_C$: solution resistance between downstream (WE2) electrode and the counter electrode, $V$: circuit potential.

By setting $R_0 = R_{ind,1} + R_{12} + 2R_C$, and combining equations 2, 5, and 6 we obtain differential equations for the dynamical evolution of the electrode potentials:

$$C_d \frac{dE_1}{dt} = \frac{V - E_1}{AR_0} - J_{F,1} + \frac{R_C}{AR_0(R_0 - 2R_C)}(E_2 - E_1) \tag{7}$$

$$C_d \frac{dE_2}{dt} = \frac{V - E_2}{AR_0} - J_{F,2} + \frac{R_C}{AR_0(R_0 - 2R_C)}(E_1 - E_2) \tag{8}$$

From Eqs. 7-8 $K_{2\to1} = K_{1\to2} = R_C/[AR_0(R_0 - 2R_C)]$.

### B. Contralateral placement configuration

At contralateral placement of reference / counter electrodes, the equivalent circuit is shown in Fig. S2. The current of the upstream electrode ($I_1$) flows in the channel to the

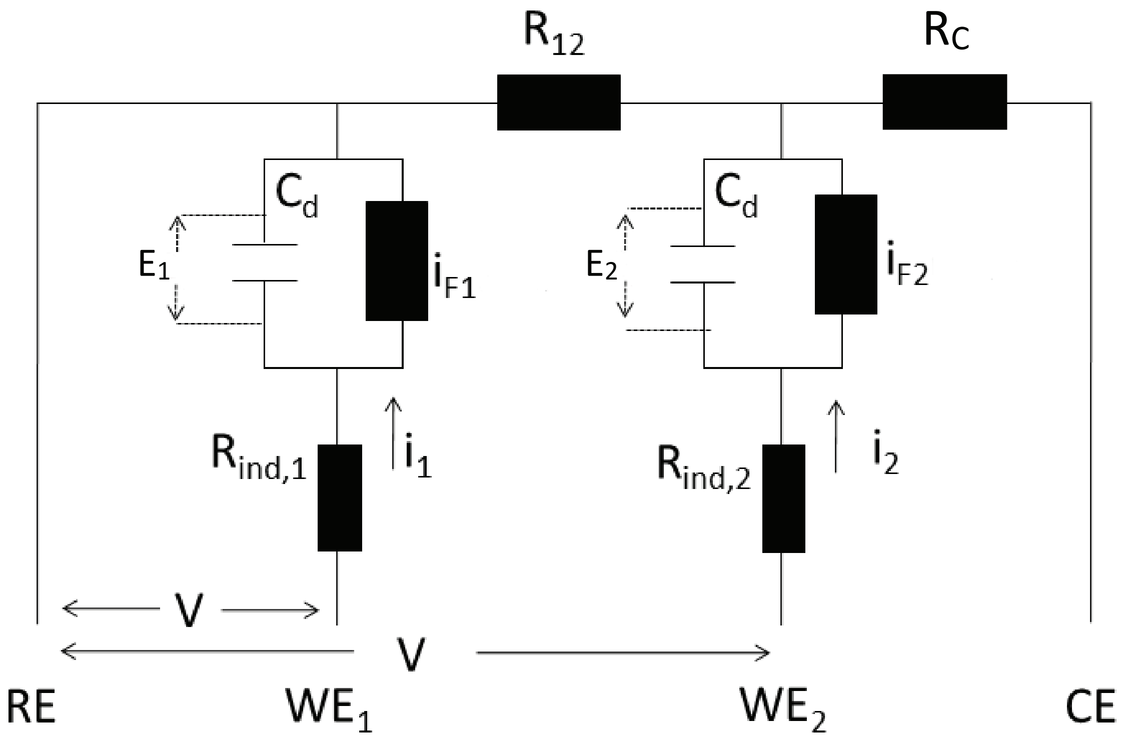


FIG. S2. The equivalent circuit of a dual-electrode electrochemical cell at contralateral placement of reference (RE) and counter (CE) electrodes, $E_{1,2}$: electrode potential, $IF_{1,2}$: Faradaic current, $C_d$: double-layer capacitance, $R_{ind,1,2}$: individual resistors, $R_{12}$: solution resistance between working electrodes, $I_1$ and $I_2$: current, $V$: circuit potential.

downstream electrode (electrode 2) through resistive circuit element $R_{12}$. The potentiostat maintains constant circuit potential $V$, therefore

For upstream electrode

$$V = I_1 R_{ind,1} + E_1 \tag{9}$$

For downstream electrode

$$V = I_2 R_{ind,2} - I_1 R_{12} + E_2 \tag{10}$$

We set $R_{ind,2} = R_{ind,1} + R_{12}$.

By rearranging equations 9 and 10

$$I_1 = \frac{V - E_1}{R_{ind,1}} \tag{11}$$

$$I_2 = \frac{V - E_2}{R_{ind,1}} - \frac{R_{12}}{R_{ind,1}(R_{ind,1} + R_{12})}(E_1 - E_2) \tag{12}$$

By setting $R_0 = R_{ind,1}$, combining equations 2, 11, and 12 we obtain differential equations for the dynamical evolution of the electrode potentials:

$$C_d \frac{dE_1}{dt} = \frac{V - E_1}{AR_0} - J_{F,1} \tag{13}$$

$$C_d \frac{dE_2}{dt} = \frac{V - E_2}{AR_0} - J_{F,2} - \frac{R_{12}}{AR_0(R_0 + R_{12})}(E_1 - E_2) \tag{14}$$

From Eqs.13-14 $K_{2\to1} = 0$, $K_{1\to2} = -R_{12}/[AR_0(R_0 + R_{12})]$

### C. Dual-reference electrode configuration

At dual-reference electrode configuration (Fig. S3), in the channel below the downstream electrode the total current ($I_1$+$I_2$) flows through resistance $R_C$. The potentiostat maintains constant circuit potential $V$, therefore

For upstream electrode

$$V = I_1 R_{ind,1} + E_1 \tag{15}$$

For downstream electrode

$$V = I_2 R_{ind,2} + (I_1 + I_2)R_C + E_2 \tag{16}$$

We set $R_{ind,2} = R_{ind,1} - 2R_C$.

By rearranging equations 15 and 16,

$$I_1 = \frac{V - E_1}{R_{ind,1}} \tag{17}$$

$$I_2 = \frac{V - E_2}{R_{ind,1}} + \frac{R_C}{R_{ind,1}(R_{ind,1} - R_C)}(E_1 - E_2) \tag{18}$$

By setting $R_0 = R_{ind,1}$ , combining equations 2, 17, and 18 we obtain differential equations for the dynamical evolution of the electrode potentials:

$$C_d \frac{dE_1}{dt} = \frac{V - E_1}{AR_0} - J_{F,1} \tag{19}$$

$$C_d \frac{dE_2}{dt} = \frac{V - E_2}{AR_0} - J_{F,2} + \frac{R_C}{AR_0(R_0 - R_C)}(E_1 - E_2) \tag{20}$$

From equations 19-20 , $K_{2\to1} = 0$, $K_{1\to2} = R_C/[AR_0(R_0 - R_C)]$.

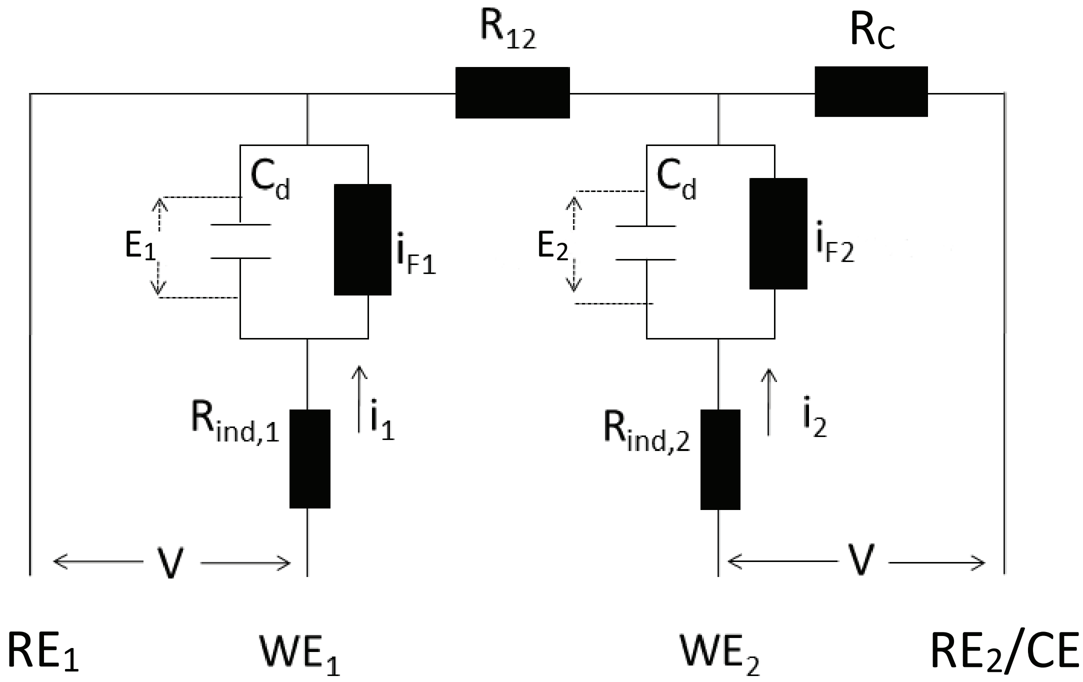


FIG. S3. The equivalent circuit of dual-reference electrode system, $E_{1,2}$: electrode potential, $IF_{1,2}$: Faradaic current, $C_d$: double-layer capacitance, $R_{ind,1,2}$: individual resistors, $R_{12}$: solution resistance between working electrodes, $I_1$ and $I_2$: current. $R_C$: solution resistance between downstream (WE2) electrode and the counter electrode, $V$: circuit potential. RE12: reference electrodes. CE: counter electrode.

### D. Switched dual-reference electrode configuration

At switched dual-reference electrode configuration (Fig. S4) the current of the upstream electrode ($I_1$) flows in the channel to the downstream electrode (electrode 2) through resistive circuit element $R_{12}$. In the channel below the downstream electrode the total current ($I_1$+$I_2$) flows through resistance $R_C$. The potentiostat maintains constant circuit potential $V$, therefore:

For upstream electrode

$$V = I_1(R_{ind,1} + R_{12}) + (I_1 + I_2)R_C + E_1 \tag{21}$$

For downstream electrode

$$V = I_2R_{ind,2} - I_1R_{12} + E_2 \tag{22}$$

We set $R_{ind,2} = R_{ind,1} + 2R_{12} + 2R_C$.

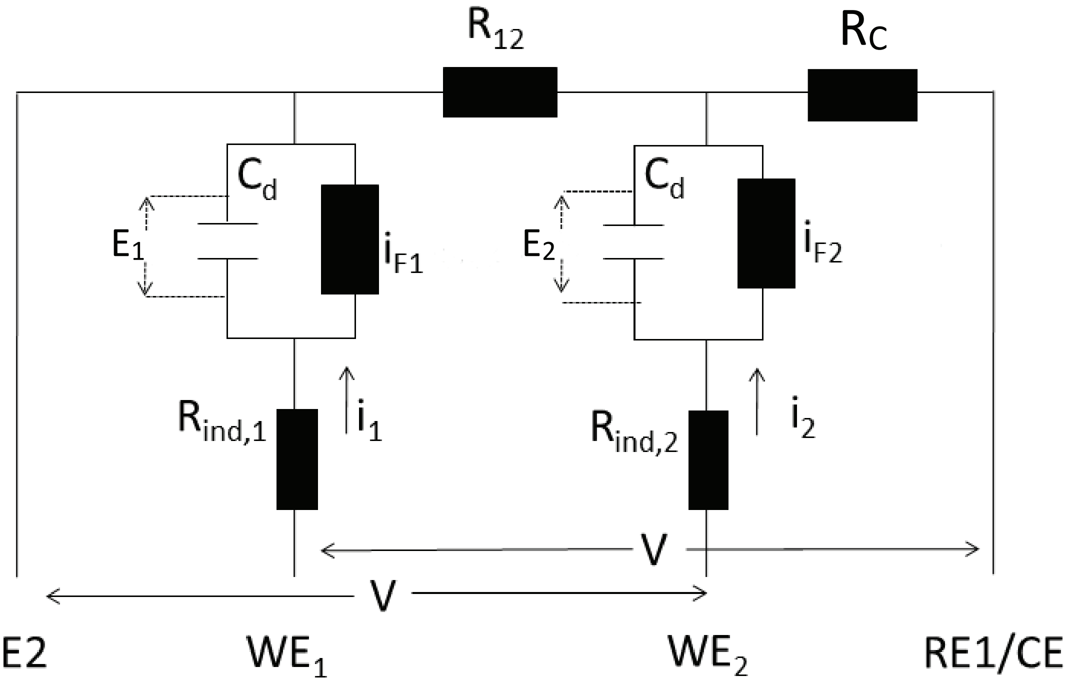


FIG. S4. The equivalent circuit of switched dual-reference electrode system, $E_{1,2}$: electrode potential, $IF_{1,2}$: Faradaic current, $C_d$: double-layer capacitance, $R_{ind,1,2}$: individual resistors, $R_{12}$: solution resistance between working electrodes, $I_1$ and $I_2$: current. $R_C$: solution resistance between downstream (WE2) electrode and the counter electrode, $V$: circuit potential. RE12: reference electrodes. CE: counter electrode.

By rearranging equations 21 and 22,

$$I_1 = \frac{V - E_1}{R_{ind,2} - R_{12}} + \frac{R_C}{(R_{ind,2} - R_{12})(R_{ind,2} - R_C)}(E_2 - E_1) \tag{23}$$

$$I_2 = \frac{V - E_2}{R_{ind,2} - R_{12}} - \frac{R_{12}}{(R_{ind,2} - R_{12})(R_{ind,2} - R_C)}(E_1 - E_2) \tag{24}$$

By setting $R_0 = R_{ind,1} + R_{12} + 2R_C$ and combining equations 2, 23, and 24 we obtain differential equations for the dynamical evolution of the electrode potentials:

$$C_d \frac{dE_1}{dt} = \frac{V - E_1}{AR_0} - J_{F,1} + \frac{R_C}{AR_0(R_0 + R_{12} - R_C)}(E_2 - E_1) \tag{25}$$

$$C_d \frac{dE_2}{dt} = \frac{V - E_2}{AR_0} - J_{F,2} - \frac{R_{12}}{AR_0(R_0 + R_{12} - R_C)}(E_1 - E_2) \tag{26}$$

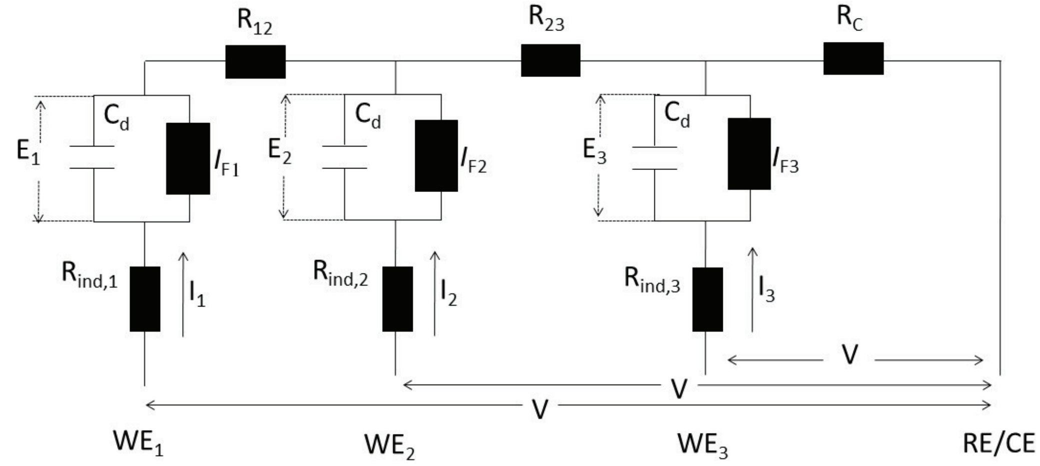


FIG. S5. The equivalent circuit of a three-working electrochemical cell at ipsilateral (traditional) placement of reference (RE) and counter (CE) electrodes, $E_{1,2,3}$: electrode potential, $IF_{1,2,3}$: Faradaic current,$C_d$: double-layer capacitance, $R_{ind,1,2,3}$: individual resistors, $R_{12}$: solution resistance between working electrodes 1 and 2, $R_{23}$: solution resistance between working electrodes 2 and 3. $I_{1,2,3}$: current. $R_C$: solution resistance between downstream (WE3) electrode and the reference electrode, $V$: circuit potential.

From equations 25-26 $K_{2\to 1} = R_C/[AR_0(R_0+R_{12}-R_C)]$, $K_{1\to 2} = -R_{12}/[AR_0(R_0+R_{12}-R_C)]$.

### E. Configuration with three working electrodes

With three working electrodes the equivalent circuit is shown in Fig. S5. The current ($I_1$) of the upstream electrode (electrode 1) flows in the channel to the downstream electrode (electrode 2) through resistive circuit element $R_{12}$. Between electrode 2 and 3, the total current ($I_1 + I_2$) flows through resistive circuit element $R_{23}$. In the channel below the

downstream electrode 3 the total current $(I_1 + I_2 + I_3)$ flows through resistance $R_C$. The potentiostat maintains constant circuit potential $V$, therefore:

For electrode 1

$$V = I_1(R_{ind,1} + R_{12}) + (I_1 + I_2)R_{23} + (I_1 + I_2 + I_3)R_C + E_1 \tag{27}$$

For electrode 2

$$V = I_2R_{ind,2} + (I_1 + I_2)R_{23} + (I_1 + I_2 + I_3)R_C + E_2 \tag{28}$$

For electrode 3

$$V = I_3R_{ind,3} + (I_1 + I_2 + I_3)R_C + E_3 \tag{29}$$

We set $R_{ind,23} = R_{ind,1} + R_{12}$, $R_{ind,3} = R_{ind,1} + R_{12} + 2R_{23}$.

By setting $R_0 = R_{ind,1} + R_{12} + 2R_{23} + 3R_C$ and rearranging equation 27-29,

$$I_1 = \frac{V - E_1}{R_0} + \frac{R_C}{(R_0 - 3R_C)}\frac{E_2 - E_1}{R_0} + \frac{R_{23}}{(R_{12} + R_{ind,1})}\frac{E_2 - E_1}{(R_0 - 3R_C)} + \frac{R_C}{(R_0 - 3R_C)}\frac{E_3 - E_1}{R_0} \tag{30}$$

$$I_2 = \frac{V - E_2}{R_0} + \frac{R_C}{(R_0 - 3R_C)}\frac{E_1 - E_2}{R_0} + \frac{R_{23}}{(R_{12} + R_{ind,1})}\frac{E_1 - E_2}{(R_0 - 3R_C)} + \frac{R_C}{(R_0 - 3R_C)}\frac{E_3 - E_2}{R_0} \tag{31}$$

$$I_3 = \frac{V - E_3}{R_0} + \frac{R_C}{(R_0 - 3R_C)}\frac{E_2 - E_3}{R_0} + \frac{R_C}{(R_0 - 3R_C)}\frac{E_1 - E_3}{R_0} \tag{32}$$

By combining equations 2 and 30-32 we obtain differential equations for the dynamical evolution of the electrode potentials:

$$C_d\frac{dE_1}{dt} = \frac{V - E_1}{AR_0} - J_{F,1} + K_{global}\left(E_2 - E_1\right) + K_{global}\left(E_3 - E_1\right) + K_{local}\left(E_2 - E_1\right) \tag{33}$$

$$C_d\frac{dE_2}{dt} = \frac{V - E_2}{AR_0} - J_{F,2} + K_{global}\left(E_1 - E_2\right) + K_{global}\left(E_3 - E_2\right) + K_{local}\left(E_1 - E_2\right) \tag{34}$$

$$C_d\frac{dE_3}{dt} = \frac{V - E_3}{AR_0} - J_{F,3} + K_{global}\left(E_2 - E_3\right) + K_{global}\left(E_1 - E_3\right) \tag{35}$$

where $K_{global} = R_C/[AR_0(R_0 - 3R_C]$, $K_{local} = R_{23}/[A(R_0 - 3R_C)(R_0 - 3R_C - 2R_{23})]$.
